# Assessment of the Content of Phenolics and Antioxidant Action of Inflorescences and Leaves of Selected Species from the Genus *Sorbus* Sensu Stricto

**DOI:** 10.3390/molecules15128769

**Published:** 2010-12-02

**Authors:** Monika A. Olszewska, Sławomira Nowak, Piotr Michel, Piotr Banaszczak, Agnieszka Kicel

**Affiliations:** 1 Department of Pharmacognosy, Faculty of Pharmacy, Medical University of Lodz, 1 Muszynski St., 90‑151 Lodz, Poland; Email: slawomira.nowak@umed.lodz.pl (S.N.); piotr.michel@umed.lodz.pl (P.M.); agnieszka.kicel@umed.lodz.pl (A.K.); 2 Arboretum, Rogow Forestry Experimental Station, Warsaw University of Life Sciences (SGGW), 95-063 Rogow, Poland; Email: arbor@wp.pl (P.B.)

**Keywords:** *Sorbus*, antioxidant activity, phenolic content

## Abstract

In order to find new sources of natural antioxidants, the antioxidant potential of 70% methanolic extracts from the inflorescences and leaves of 16 species from the genus *Sorbus* s.s. was evaluated using two complementary *in* *vitro* test systems: the DPPH (2,2-diphenyl-1-picrylhydrazyl) radical-scavenging assay and the AAPH [2,2′-azobis-(2-amidinopropane)dihydrochloride]-induced linoleic acid (LA) peroxidation test. The radical-scavenging capacities of the extracts towards the DPPH radical were in the range of 0.25−0.86 millimolar Trolox^®^ equivalents/g dry weight. They were significantly correlated (*r* = -0.8089, *p* < 0.001) with the results of the LA-peroxidation test, indicating the *Sorbus* extracts to be universal antioxidants. Significant linear correlations were also found between the different antioxidant potentials and total phenolic contents as estimated by the Folin-Ciocalteu method and further verified by serial determinations of proanthocyanidins, chlorogenic acid isomers and flavonoids (|*r*| in the range of 0.71−0.95, *p* < 0.001). Cluster analysis of the data matrix identified the ten samples (inflorescences of *S. aucuparia*, *S. pohuashanensis*, *S. decora*, *S. koehneana*, *S. commixta*, *S. gracilis*, and *S. sitchensis*, and the leaves of *S. wilfordii*, *S. pogonopetala*, and *S. gracilis*) exhibiting the highest antioxidant activity and total phenolic levels and therefore the greatest potential as effective sources for natural health products.

## 1. Introduction

The large genus *Sorbus* sensu stricto (Rosaceae, Maloideae) is confined to the Northern Hemisphere and has a circumboreal distribution, being commonly found in Europe, North Africa, Asia and northern North America [1]. The majority of the over 70 species included in the genus have not been phytochemically characterised, except for a few species with known ethnomedical significance such as *S. aucuparia* L., *S. americana* Marsh., *S. cashmiriana* Hedl., *S. commixta* Hedl., and *S. decora* (Sarg.) C.K. Schneid. These plants are reported to have antioxidative [2,3,4,5], anti-atherogenic [6], anti-inflammatory [7], anti-diabetic [4], vasoprotective and vasorelaxant activities [8]. Most of these bioactivities can be explained by the presence of phenolic constituents. Indeed, a direct correlation between high antioxidant capacity and total phenolic content has been found for numerous species representing the *Sorbus* genera sensu stricto (s.s.) [2,3] and sensu lato (s.l.) [9,10]. The known *Sorbus* phenolics range from simple phenolic acids, their esters with quinic acid (mostly caffeoylquinic acids), flavonols (conjugates of quercetin, kaempferol, and sexangularetin), anthocyanins (cyanidin and pelargonidin derivatives), and tannin-type proanthocyanidins [2,3,10].

Phenolic compounds are recognised as beneficial to human health and disease prevention, mostly due to their antioxidant activity and ability to neutralize reactive oxygen species (ROS). Under oxidative stress conditions, ROS can react with important biomolecules, causing cellular injury and the development of chronic diseases, such as atherosclerosis, coronary diseases, cancer, and neurodegenerative brain disorders. To ensure the optimum protection from the over-production of ROS, there is a need to provide antioxidants as part of the diet [11]. Antioxidants are also required to preserve food for prolonged storage and transport [11]. Food and pharmaceutical products are thus normally supplemented with synthetic phenolic antioxidants such as butylated hydroxyanisole (BHA) or butylated hydroxytoluene (BHT). However, natural plant phenolics may be more effective at reducing ROS levels compared to single synthetic compounds due to the synergistic actions of a wide range of active molecules existing in plant products [11]. In addition, the dietary intake of synthetic antioxidants could cause genotoxicity and carcinogenicity at high concentrations [11]. On the other hand, many phenolic constituents of herbal medicines and dietary plants have been identified as safe and potent antioxidants, functioning as free radical scavengers, reducing agents and chelators of transition metal ions [12]. The search for rich sources of natural antioxidants has, therefore, received much attention, and efforts have been made to identify plant extracts or compounds capable of replacing synthetic ones [13,14].

Although a variety of plant materials (herbs, leaves, flowers, fruits, vegetables, *etc*.) have been studied for antioxidant activity and phenolic content, only a few have been found to be sufficiently rich in phenolics to be used for the cost-effective production of natural antioxidants [13,14]. An ideal antioxidant plant material would fulfil several criteria. These include mainly potent antioxidant action in various test systems (universal chemistry), a high level of total phenolics (not less than 8–10% dry weight (dw) of gallic acid equivalents, GAE), and low toxicity.

The results of previous studies of the genus *Sorbus* s.l. revealed that several species are promising sources of powerful antioxidants [2,3,4,5,9,10]. Significantly high total phenolic contents have been found in the inflorescences (11.83% dw GAE) and leaves (9.09% dw GAE) of *S. aucuparia*, and were correlated with the universal, potent antioxidant capacities of the appropriate extracts as measured by three different *in vitro* test systems [3]. It is noteworthy that the levels of phenolics and antioxidant activities found for *S. aucuparia* (the model species for the genus *Sorbus* s.s.) were higher than those observed for *S. aria*, *S. intermedia* and *S. torminalis* (species representing subgenera *Aria* and *Torminaria* from the genus *Sorbus* s.l.) [3,10]. However, there have been no prior reports on the antioxidant activity and phenolic levels of inflorescences and leaves of any species from the genus *Sorbus* s.s. with the exception of *S. aucuparia*.

Thus, the aim of this project was to screen the inflorescences and leaves of 16 selected species from the genus *Sorbus* s.s. (Table 1), including *S. aucuparia*, with respect to their phenolic profile and antioxidant activity. The plant extracts were assayed using two complementary *in vitro* test systems: the DPPH free radical-scavenging method and the AAPH-induced linoleic acid peroxidation test. To identify the compounds responsible for the tested activity, the quantitative phenolic profiles of the plant materials were monitored by HPLC and by UV-photometric methods, and the relationship between the antioxidant capacity and the phenolic content was investigated. Finally, all data were subjected to hierarchical cluster analysis to distinguish the group of species and plant materials with the greatest potential as valuable sources of natural antioxidants and candidates for *in vivo* studies of antioxidant protection.

**Table 1:** Antioxidant activity of the studied *Sorbus* species as measured by the DPPH free radical‑scavenging assay and LA-peroxidation test.

Sample No.	Plant sources		Antioxidant activity ^b^		
	Scientific name	Plant part tested^ a^	DPPH EC_50_		LA peroxidation (% inhibition) ^e^
			(µg/mL) ^c^	TEAA (mmol/g) ^d^	
1.	*Sorbus aucuparia* L.	I	16.69 ± 0.26 ^A, B^	0.78	68.34 ± 0.59 ^A^
2.	*Sorbus aucuparia* L.	L	24.10 ± 0.29 ^C^	0.54	58.69 ± 0.46 ^B, C^
3.	*Sorbus pohuashanensis* (Hance) Hedl.	I	17.89 ± 0.55 ^B, D^	0.73	68.69 ± 1.24 ^A^
4.	*Sorbus pohuashanensis* (Hance) Hedl.	L	43.86 ± 1.51 ^E^	0.30	50.21 ± 0.96 ^D^
5.	*Sorbus scalaris* Koehne	I	27.65 ± 1.09 ^F^	0.47	55.23 ± 1.46 ^E^
6.	*Sorbus scalaris* Koehne	L	57.86 ± 1.63 ^G^	0.23	41.70 ± 1.05 ^F^
7.	*Sorbus prattii* Koehne var. *prattii*	L	24.26 ± 0.45 ^C^	0.54	57.51 ± 0.87 ^B^
8.	*Sorbus americana* Marsh.	L	38.76 ± 0.84 ^H^	0.34	54.29 ± 0.85 ^E^
9.	*Sorbus commixta* Hedl.	I	23.22 ± 0.33 ^C^	0.56	78.21 ± 1.61 ^G^
10.	*Sorbus commixta* Hedl.	L	28.56 ± 0.64 ^F^	0.46	58.65 ± 1.28 ^B, C^
11.	*Sorbus decora* (Sarg.) C.K. Schneid.	I	16.20 ± 0.27 ^A^	0.81	70.99 ± 1.39 ^H^
12.	*Sorbus decora* (Sarg.) C.K. Schneid.	L	27.21 ± 0.72 ^F^	0.48	59.99 ± 0.97 ^C^
13.	*Sorbus wilfordii* Koehne	L	15.23 ± 0.54 ^A^	0.86	86.92 ± 1.15 ^I^
14.	*Sorbus sambucifolia* (Cham. & Schltdl.) M. Roem.	I	28.03 ± 0.80 ^F^	0.47	58.12 ± 1.50 ^B, C^
15.	*Sorbus sambucifolia* (Cham. & Schltdl.) M. Roem.	L	52.63 ± 1.25 ^I^	0.25	54.03 ± 1.05 ^E^
16.	*Sorbus gracilis* (Sieb. & Zucc.) K. Koch	I	19.09 ± 0.69 ^D, J^	0.68	73.01 ± 1.09 ^J^
17.	*Sorbus gracilis* (Sieb. & Zucc.) K. Koch	L	20.71 ± 0.66 ^J^	0.63	70.72 ± 1.00 ^H^
18.	*Sorbus sitchensis* M. Roem.	I	20.75 ± 0.40 ^J^	0.63	68.26 ± 0.78 ^A^
19.	*Sorbus sitchensis* M. Roem.	L	54.23 ± 1.08 ^K^	0.24	53.13 ± 0.86 ^E^
20.	*Sorbus cashmiriana* Hedl.	L	48.59 ± 1.05 ^L^	0.27	53.59 ± 0.78 ^E^
21.	*Sorbus koehneana* C.K. Schneid.	I	16.20 ± 0.52 ^A^	0.81	73.34 ± 1.07 ^J^
22.	*Sorbus koehneana* C.K. Schneid.	L	24.74 ± 0.34 ^C^	0.53	54.15 ± 0.94 ^E^
23.	*Sorbus pogonopetala* Koehne	L	19.87 ± 0.48 ^J^	0.66	74.73 ± 1.13 ^J^
24.	*Sorbus setschwanensis* (C.K. Schneid.) Koehne	L	23.30 ± 0.56 ^C^	0.56	63.77 ± 0.70 ^K^
Quercetin			1.44 ± 0.05		88.38 ± 1.15
Trolox			3.27 ± 0.10		92.74 ± 1.10

^a^ I, inflorescences; L, leaves. ^b^ Results are mean values of triplicate analyses ± S.D. Different superscripts (capitals) in each column indicate significant differences in the mean values at *p* < 0.05. ^c, d^ Scavenging efficiency (amount of antioxidant needed to decrease the initial DPPH concentration by 50%) expressed as follows: ^c ^in µg of dry plant material or the standard/mL of DPPH solution; ^d ^in millimolar Trolox^®^ antioxidant equivalents (TEAA)/g of dry plant material. ^e ^Inhibition ratio of LA-peroxidation after incubation with the final antioxidant concentration of 1.50 mg/mL for the plant materials or 0.20 mg/mL for the standards.

## 2. Results and Discussion

### 2.1. Phenolic profile of the analysed Sorbus tissues

The total phenolic content (TPC) of the *Sorbus* extracts was determined by the Folin-Ciocalteu (FC) photometric assay. In this method, a mixture of phosphotungstic and phosphomolybdic acids is reduced under alkaline conditions to blue oxides of tungsten and molybdenum upon phenol oxidation [15]. The reaction is commonly used to obtain a crude estimate of TPC as gallic acid equivalents (GAE), but it can also be considered as an indirect measure of antioxidant activity because of the basic redox mechanism, similar to that occurring in other antioxidant methods [11,15].

As shown in Table 2, the majority of the tested *Sorbus* tissues exhibited high levels of TPC ranging from 8.08 to 12.31% dw of GAE, with the average value for all samples of 8.81% GAE, and with the highest content found for the leaves of *S. wilfordii* (sample **13**, 12.31 ± 0.32% GAE). In ten samples (**1**, **3**, **11**, **13**, **16**–**18**, **21**, **23**, **24**) the TPC was not less than 10% GAE. For different plant tissues derived from the same *Sorbus* species, the TPC level was remarkably higher in the inflorescences than in the leaves. The single exception was *S. gracilis*, whose inflorescences and leaves (samples **16** and **17**) exhibited similar but still statistically different TPC values (11.06 and 10.72% GAE, *p* < 0.05).

It is evident that the TPC value determined by the FC assay does not give a full picture of the real phenolic constituents in plant extracts. Thus, for verification of the phenolic levels in *Sorbus*, further determinations of the main phenolic groups were performed.

**Table 2:** Total phenolic content, total content of chlorogenic acid isomers, and total proanthocyanidin content in *Sorbus* extracts.^a^

Sample	No.	Total phenols (GAE, %)^ b^	Caffeoylquinic acids ^c^ (%)		Proanthocyanidins (CyE, %) ^d^
			NChA	ChA	
**CL1:**	1.	10.02 ± 0.22 ^A^	0.74 ± 0.01 ^A^	2.27 ± 0.01 ^A^	5.94 ± 0.12 ^A^
	3.	11.32 ± 0.03 ^B^	0.70 ± 0.01 ^B^	2.48 ± 0.02 ^B, C^	7.67 ± 0.05 ^B^
	17.	10.72 ± 0.16 ^C^	0.03 ± 0.01 ^C^	0.93 ± 0.01 ^D, E^	6.56 ± 0.07 ^C^
	18.	10.08 ± 0.11 ^A, D^	0.45 ± 0.02 ^D^	3.13 ± 0.06 ^F^	7.14 ± 0.11 ^D^
	11.	11.67 ± 0.16 ^E^	1.26 ± 0.02 ^E^	3.85 ± 0.06 ^G^	6.40 ± 0.12 ^C^
	21.	11.67 ± 0.05 ^E^	1.98 ± 0.01 ^F^	2.05 ± 0.02 ^H^	6.86 ± 0.12 ^E^
	16.	11.06 ± 0.16 ^F^	0.19 ± 0.01 ^G^	3.31 ± 0.03 ^I^	6.54 ± 0.16 ^C^
	23.	10.90 ± 0.26 ^C, F^	0.22 ± 0.01^ H^	1.63 ± 0.02 ^J^	5.89 ± 0.04 ^A ^
	9.	9.29 ± 0.14 ^G^	0.76 ± 0.01 ^A^	3.92 ± 0.02 ^G^	5.98 ± 0.21 ^A^
	13.	12.31 ± 0.32 ^H^	0.13 ± 0.01 ^I^	2.58 ± 0.02 ^B^	5.31 ± 0.04 ^F^
**CL2:**	2.	8.23 ± 0.06 ^I, J^	0.51 ± 0.01 ^J^	1.90 ± 0.08 ^K^	3.59 ± 0.09 ^G, H^
	7.	8.44 ± 0.08 ^J^	1.10 ± 0.04 ^K^	2.81 ± 0.09 ^L^	2.90 ± 0.06 ^I^
	10.	8.08 ± 0.25 ^I^	0.05 ± 0.01 ^C^	0.79 ± 0.01 ^E, M^	3.58 ± 0.09 ^G, H^
	12.	8.10 ± 0.09 ^I^	0.19 ± 0.01 ^G^	2.10 ± 0.01 ^H^	4.03 ± 0.09 ^J^
	14.	8.22 ± 0.40 ^I, J^	0.42 ± 0.01 ^L^	4.17 ± 0.12 ^N^	3.79 ± 0.16 ^H, K^
	5.	8.47 ± 0.21 ^J^	0.60 ± 0.01 ^M^	2.36 ± 0.02 ^A, C^	5.68 ± 0.10 ^L, M^
	22.	9.87 ± 0.11 ^A^	0.53 ± 0.01 ^J^	1.97 ± 0.02 ^H, K^	5.81 ± 0.09 ^A, M^
	24.	10.18 ± 0.17^ D^	0.22 ± 0.01 ^H^	2.61 ± 0.02 ^B^	5.56 ± 0.08 ^L^
**CL3:**	4.	6.26 ± 0.19 ^K^	0.12 ± 0.01 ^I, O^	0.67 ± 0.01 ^M, O^	3.93 ± 0.03 ^J, K^
	20.	5.78 ± 0.12 ^L^	0.37 ± 0.01 ^N^	1.25 ± 0.01 ^P^	4.02 ± 0.05 ^J^
	8.	6.47 ± 0.09 ^K^	0.04 ± 0.01 ^C^	1.85 ± 0.01 ^K^	3.66 ± 0.09 ^G^
	6.	4.23 ± 0.15 ^M^	0.36 ± 0.01 ^N^	1.24 ± 0.01 ^P^	1.47 ± 0.04 ^N^
	15.	5.07 ± 0.02 ^N^	0.10 ± 0.02 ^O^	1.02 ± 0.01 ^D^	1.96 ± 0.09 ^O^
	19.	4.89 ± 0.07 ^N^	0.05 ± 0.01 ^C^	0.56 ± 0.01 ^O^	1.48 ± 0.05 ^N^

^a ^Results are mean values of triplicate analyses calculated per dw of the plant material ± S.D. Different superscripts (capitals) in each column indicate significant differences in the mean values at *p* < 0.05. Sample codification as in Table 1. CL1–CL2, groups obtained after cluster analysis. ^b ^Total phenolic content expressed in GAE, gallic acid equivalents. ^c ^Content of chlorogenic acid isomers quantified by HPLC: NChA, neochlorogenic acid; ChA, chlorogenic acid. ^d ^Total proanthocyanidin content expressed in CyE, cyanidin chloride equivalents.

As shown in Table 2 and Table 3, the predominant components in the majority of the extracts were proanthocyanidins, followed by caffeoylquinic acids and flavonoids. In three samples (**6**, **7**, and **14**), the sum of caffeoylquinic acid isomers (chlorogenic (ChA) and neochlorogenic (NChA) acids) was greater. The tested plant materials showed a wide range of total proanthocyanidin content (1.47−7.67% dw calculated as cyanidin equivalents, CyE), sum of caffeoylquinic acid isomers (0.61−5.11% dw), and total content of flavonoid aglycones (0.121−1.322% dw). 

**Table 3:** Total content of flavonoid aglycones in *Sorbus* extracts.^a^

Sample	No.	Flavonoid aglycones ^d^ (%)		
		QU	KA	SX
**CL1:**	1.	1.048 ± 0.034 ^A^	0.084 ± 0.002 ^A^	0.190 ± 0.002 ^A^
	3.	0.400 ± 0.014 ^B^	0.039 ± 0.001 ^B^	0.017 ± 0.001 ^B^
	17.	0.113 ± 0.001 ^C^	0.008 ± 0.001 ^C^	nd
	18.	0.384 ± 0.028 ^B, K^	0.021 ± 0.001 ^D^	0.048 ± 0.001 ^C^
	11.	0.839 ± 0.008 ^D, E^	0.059 ± 0.001 ^E, F^	0.070 ± 0.002 ^D^
	21.	0.266 ± 0.014 ^F^	0.024 ± 0.003 ^D^	0.050 ± 0.005 ^C^
	16.	0.194 ± 0.006 ^G, H^	0.012 ± 0.001 ^C^	0.072 ± 0.003 ^D^
	23.	0.382 ± 0.023 ^B, K^	0.263 ± 0.006 ^G^	nd
	9.	0.422 ± 0.008 ^B, J^	0.050 ± 0.003 ^F^	0.045 ± 0.003 ^C^
	13.	0.878 ± 0.015 ^E, I^	0.046 ± 0.001 ^B, F^	nd
**CL2:**	2.	0.903 ± 0.003 ^I^	0.157 ± 0.005^ H^	nd
	7.	0.790 ± 0.020 ^D^	0.117 ± 0.001 ^I, J^	nd
	10.	0.470 ± 0.004 ^J^	0.011 ± 0.001 ^C^	nd
	12.	0.474 ± 0.012 ^J^	0.035 ± 0.001 ^B^	nd
	14.	0.805 ± 0.017 ^D^	0.058 ± 0.001 ^E, F^	0.127 ± 0.003 ^E^
	5.	0.341 ± 0.005 ^K^	0.062 ± 0.001 ^E^	0.145 ± 0.002 ^F^
	22.	0.247 ± 0.007 ^F, G^	0.106 ± 0.002 ^K^	nd
	24.	0.566 ± 0.031 ^L^	0.306 ± 0.007 ^L^	nd
**CL3:**	4.	0.123 ± 0.004 ^C, H^	0.030 ± 0.001 ^B, D^	nd
	20.	0.525 ± 0.006 ^L^	0.113 ± 0.002 ^J, K^	nd
	8.	0.460 ± 0.019 ^J^	0.039 ± 0.001 ^B^	nd
	6.	0.217 ± 0.009 ^G^	0.125 ± 0.009 ^I^	nd
	15.	0.163 ± 0.006 ^C, H^	0.011 ± 0.001 ^C^	nd
	19.	0.270 ± 0.012 ^F^	0.023 ± 0.001 ^D^	nd

^a ^Results are mean values of triplicate analyses calculated per dw of the plant material ± S.D. Different superscripts (capitals) in each column indicate significant differences in the mean values at *p* < 0.05. Sample codification as in Table 1. CL1–CL2, groups obtained after cluster analysis. ^b ^Content of flavonoid aglycones quantified by HPLC: QU, quercetin; KA, kaempferol; SX, sexangularetin; nd – not detected.

The highest levels of proanthocyanidins were found for the inflorescences of *S. pohuashanensis* (sample **3**, 7.67 ± 0.05% CyE) and *S. sitchensis* (sample **18**, 7.14 ± 0.11% CyE). The highest content of caffeoylquinic acid isomers was found in the inflorescences of *S. decora* (sample **11**, 5.11 ± 0.08%). With the exception of sample **21** (inflorescences of *S. koehneana*), the dominant caffeoylquinic isomer in all other tested samples was ChA, with the levels constituting 71−97% of the sum of both quantified isomers. In sample **21**, the levels of ChA and NChA were similar and reached about 2% dw each. Of the flavonoids, quercetin was the predominant aglycone with the levels varying widely among the assayed samples (0.113−1.048% dw). Quercetin was accompanied by kaempferol (0.011−0.306% dw) in the leaf samples, and by kaempferol (0.021−0.084% dw) and sexangularetin (0.017−0.190% dw) in the inflorescences. The highest flavonoid content was found for the inflorescences of *S. aucuparia* (sample **1**, 1.322% dw).

For the majority of samples, the total content of the particular phenolic groups (TPh, proanthocyanidins + caffeoylquinic acids + flavonoid aglycones) was consistent with the TPC level expressed in GAE, which is in accordance with the results of our earlier study of three *Sorbus* species [3]. This correlation was statistically significant at *p* < 0.001, and was characterised by high correlation and determination coefficients *r* (*R^2^*) = 0.8551 (0.7312). Remarkable differences in these contents were observed only for six leaf samples (**10**, **13**, **15**, **17**, **19**, and **23**), in which the TPC values were significantly higher than the TPh levels. This discrepancy could be caused by the presence of some unquantified phenolic metabolites, such as simple phenolic acids, or by the presence of some interfering substances that are reactive in the FC assay, such as sugars or ascorbic acid [11,15]. If the six above-mentioned cases were excluded, the correlation between TPC and TPh levels was especially high (*r* (*R^2^*) = 0.9181 (0.8429), *p <* 0.001). This is clear evidence that the listed groups of phenolic compounds are the main phenolic metabolites present in the tested *Sorbus* samples and that they are also the most important phenolic determinants of the redox activity of these samples.

### 2.2. Antioxidant activity of the extracts from Sorbus tissues

Given the differences in the basic reaction mechanisms among the wide number of available test systems, an approach involving at least two different assays is highly advisable to fully characterise the antioxidant properties of plant extracts [11,15]. On this basis, the *in vitro* antioxidant activity of *Sorbus* tissues was assayed using two complementary tests: the DPPH free radical scavenging method (the widely used system involving the single electron transfer (SET) reaction) and inhibition of the AAPH-induced linoleic acid (LA) peroxidation test (involving the hydrogen atom transfer (HAT) mechanism, a more physiologically and food-relevant system).

The antioxidant results are summarised in Table 1. A significant (*p <* 0.001) correlation was found between the results of the two methods, characterised by the coefficients of correlation (*r* = -0.8089) and determination (*R^2^* = 0.6543), suggesting that the analysed *Sorbus* extracts utilise both the HAT and SET reaction mechanisms. Although these *R*-values are both quite high, they were lower than those observed for the correlations between the DPPH and FC assay and also between the LA-peroxidation test and the FC method (Table 4).

**Table 4:** Correlation (*r*) and determination (*R^2^*) coefficients between different parameters of antioxidant capacity of *Sorbus* extracts and the content of phenolic compounds.

*r* (*R^2^*)	DPPH EC_50_ (µg/mL)^ c^	LA peroxidation (% inhibition)^ c^
TPC (GAE, %)^ a^	-0.9535 (0.9092)*	0.8664 (0.7506)*
TPh (%)^b^	-0.8525 (0.7267)*	0.7377 (0.5442)*
Proanthocyanidins (CyE, %)	-0.8007 (0.6411)*	0.7456 (0.5559)*
Caffeoylquinic acids (ChA+NChA, %)	-0.6238 (0.3891)**	0.4998 (0.2498)***
Flavonoid aglycones (%)	-0.4248 (0.1804)***	0.2297 (0.0528)

^a ^TPC, total phenolic content determined by the FC method expressed in GAE, gallic acid equivalents. ^b^ TPh, total phenolic content expressed as the sum of proanthocyanidins, caffeoylquinic acids and flavonoid aglycones. ^c^ Significance levels: *p < 0.001; **p < 0.01; ***p < 0.05.

**Figure 1:**
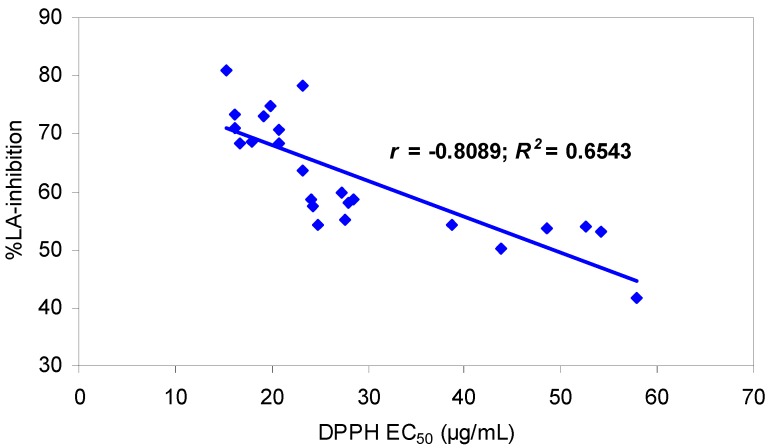
Scatter diagram of the correlation between parameters of antioxidant capacity of *Sorbus* extracts measured by the DPPH test and LA-peroxidation assay.

This lower correlation might be attributed to the different reaction mechanisms and was also observed for other plant extracts [16]. Analysis of the scatter plot (Figure 1) for the DPPH and LA-peroxidation tests showed that the samples exhibit differences in antioxidant results between the two assay methods and that they could be distributed in three main groups. This hypothesis was verified statistically by partitional clustering (*k*-means method). The first cluster (CL1) grouped ten samples (**1**, **3**, **17**, **18**, **11**, **21**, **16**, **23**, **9**, and **13**) exhibiting the highest antioxidant capacities in both systems with a mean scavenging efficiency for the DPPH radical of EC_50_ = 18.56 ± 2.57 μg/mL (TEAA = 0.71 ± 0.09 mmol/g) and a mean LA-inhibition in the peroxidation test of 72.73 ± 4.28%. The eight samples grouped in the second cluster CL2 (**2**, **7**, **10**, **12**, **14**, **5**, **22**, and **24**) demonstrated relatively high scavenging capacities (mean EC_50_ = 25.98 ± 2.08 μg/mL, TEAA = 0.51 ± 0.04 mmol/g) and moderate inhibition of LA peroxidation (mean value 58.26 ± 2.94%). The samples with the lowest antioxidant activity (**4**, **8**, **20**, **6**, **15**, and **19**) constituted the third cluster (CL3), with a mean EC_50_ value of 49.32 ± 7.07 μg/mL (TEAA = 0.26 ± 0.04 mmol/g) and a mean inhibition of LA-peroxidation of 51.16 ± 4.86%.

There are limited data in the literature [13,14] with which it would be possible to easily compare our antioxidant results, given the differences in the assay protocols. According to these systematic screening studies, only about 20% of known plant materials have high or extremely high antioxidant activity as measured by the DPPH method, and exhibit TEAA values in the range 0.25−1.00 mmol/g dw (14% of plants) or even higher (6%). As shown in Table 1, all analysed *Sorbus* samples were found to have high (clusters CL2 and CL3) and very high antioxidant activity (cluster CL1). It is noteworthy that the majority of plant tissues previously reported to have extremely high antioxidant activity (TEAA ≥ 1.00 mmol/g dw), such as gum of *Acacia catechu*, seeds of *Magnifera indica*, bark of *Myrica nagi*, pericarp of *Punica granatum*, and galls of *Rhus succedanea*, contained hydrolysable tannins (gallotaninns) as the main active components [14]. Gallotannins are known as potent scavengers of the DPPH radical [17], but they could also be hepatotoxic in internal applications due to the hydrolytic liberation of gallic acid [18]. As described in Section 2.1, the main antioxidant constituents of the studied *Sorbus* plants are proanthocyanidins, isomers of chlorogenic acid and flavonols (quercetin derivatives), compounds that are considered to be safe in rational therapy [19,20,21]. Potential safety issues exist only if megadoses of these phenolics are consumed daily [19,20]. Therefore, with their high activity and high total phenolic content, the analysed *Sorbus* tissues appear to be very promising and potent sources of safe natural antioxidants.

### 2.3. Relationships among the estimates of antioxidant capacities obtained from the DPPH and LA-peroxidation assays, and phenolic contents

A linear regression analysis was performed for all experimental data (*n* = 24) to study the relationships among the estimated values of antioxidant activity and phenolic content. The results (Table 4) revealed a strong and significant correlation ((|*r*| > 0.86, *p* < 0.001) between the antioxidant results obtained by the two methods and the total phenolic content measured by the FC method.

Thus, the levels of TPC expressed in GAE could be considered to be an important indicator of antioxidant capacity, and may be applied to preliminarily screen plant extracts for use as natural sources of antioxidants. The high correlation observed between the results of both antioxidant tests and the total phenolic content expressed as the sum of proanthocyanidins, caffeoylquinic acids and flavonoids (TPh) confirmed that these phenolic groups were synergistic determinants of the tested activity. Of the three analysed groups, proanthocyanidins could be considered to be primarily responsible for the estimated antioxidant activity, followed by the chlorogenic acid isomers and flavonoid aglycones. These results are in accordance with published reports on the potent activity of proanthocyanidins, chlorogenic acid, and flavonoids (especially quercetin derivatives) in numerous antioxidant *in vitro* test systems [17,22,23]. On the other hand, the observed low impact of flavonoids on the antioxidant activity conflicts with the results of our earlier study of *Sorbus* species [3]. This discrepancy could be explained by the low number of samples (*n* = 9) and close genetic relationship between the three species tested previously (two parental and their descendant hybrid species). It is also known that the antioxidant activity of flavonoids is strongly structurally dependent [17], and thus structural differences between the real flavonoid constituents, which exist in the *Sorbus* extracts mostly in the form of glycosides [10], and the aglycones estimated after acid hydrolysis, could bring about the low correlation found in the present study.

### 2.4. Hierarchical cluster analysis of the Sorbus samples

**Figure 2:**
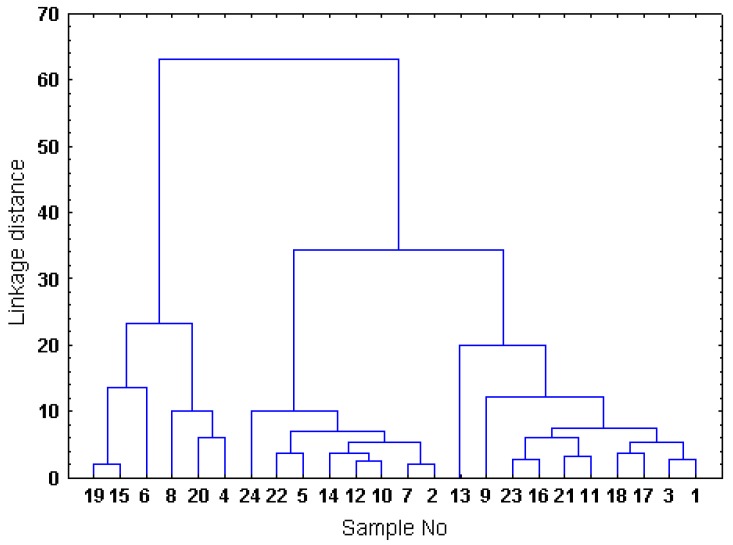
Dendrogram built by hierarchical cluster analysis with all of the variables using the complete linkage method and Euclidean squared distance. Codification is given in Table 1.

An agglomerative hierarchical cluster analysis (CA) was performed for all of the experimental data, taking the Euclidean distance as the metric and the complete linkage method as the amalgamation rule. The results were in accordance with those obtained by partitional clustering (Section 2.2). As presented in the dendrogram (Figure 2), three main clusters were found, corresponding to the three levels of antioxidant capacities and phenolic contents of the analysed *Sorbus* samples.

The ten samples that grouped in the cluster CL1 (inflorescences of *S. aucuparia*, *S. pohuashanensis*, *S. decora*, *S. koehneana*, *S. commixta*, *S. gracilis*, and *S. sitchensis*, as well as the leaves of *S. wilfordii*, *S. pogonopetala*, and *S. gracilis*) were characterised by the highest antioxidant capacities (see section 2.2), TPC level (mean TPC = 10.90 ± 0.91% GAE), total proanthocyanidin contents (mean level = 6.43 ± 0.69% CyE) and contents of caffeoylquinic acids (mean level = 3.26 ± 1.24%). Therefore, these samples could be considered to have the greatest potential as valuable and cost-effective sources of natural phenolic antioxidants among the studied *Sorbus* samples.

By converting the original average value of EC_50_ for the DPPH test (18.76 ± 2.65 μg/mL) to μg phenolics/mL using the mean TPC value for the cluster CL1, we obtain the recalculated value of EC_50_ = 2.05 μg phenolics/mL.

The standards of quercetin and Trolox^®^ assayed simultaneously exhibited EC_50_ values of 1.44 ± 0.05 and 3.27 ± 0.10 μg/mL, respectively. According to the literature, the antioxidant activity of quercetin and trolox is remarkably higher than that of the synthetic antioxidants that are used industrially, such as BHA and BHT [24,25]. We believe that this is convincing evidence of the potent antioxidant activity of the ten *Sorbus* samples grouped in the cluster CL1. Thus, these samples appear to be good candidates for *in vivo* studies of antioxidant protection with high potential for use as possible substitutes for the currently used synthetic antioxidants.

## 3. Experimental

### 3.1. Plant material

Samples of inflorescences and leaves of the 16 studied *Sorbus* species were collected and authenticated in June 2009 in the Arboretum (51°49′N, 19°53′E), Forestry Experimental Station of Warsaw University of Life Sciences (SGGW) in Rogów (Poland). Voucher specimens (Table 5) were deposited in the herbarium of the Department of Pharmacognosy, Medical University of Łódź, Poland. The studied samples are listed in Table 1.

**Table 5:** Voucher specimen numbers of the studied *Sorbus* species.

Scientific name	Voucher specimen no.	Scientific name	Voucher specimen no.
*Sorbus aucuparia* L.	KFG/HB/07001-SAUC	*Sorbus sambucifolia* (Cham. & Schltdl.) M. Roem.	KFG/HB/08008-SSAM
*Sorbus pohuashanensis* (Hance) Hedl.	KFG/HB/07002-SPOH		
*Sorbus scalaris* Koehne	KFG/HB/08005-SSCA	*Sorbus gracilis* (Sieb. & Zucc.) K. Koch	KFG/HB/07006-SGRA
*Sorbus prattii* Koehne var. *prattii*	KFG/HB/08006-SPRA	*Sorbus sitchensis* M. Roem.	KFG/HB/08009-SSIT
*Sorbus americana* Marsh.	KFG/HB/07003-SAME	*Sorbus cashmiriana* Hedl.	KFG/HB/07007-SCAS
*Sorbus commixta* Hedl.	KFG/HB/07004-SCOM	*Sorbus koehneana* C.K. Schneid.	KFG/HB/07008-SKOE
*Sorbus decora* (Sarg.) C.K. Schneid.	KFG/HB/08007-SDEC	*Sorbus pogonopetala* Koehne	KFG/HB/07009-SPOG
*Sorbus wilfordii* Koehne	KFG/HB/07005-SWIL	*Sorbus setschwanensis* (C.K. Schneid.) Koehne	KFG/HB/08010-SSET

### 3.2. Chemicals and instrumentation

HPLC or GC grade purity reagents and standards, such as 2,2-diphenyl-1-picryl hydrazyl (DPPH); 2,2′-azobis-(2-amidinopropane) dihydrochloride (AAPH); (±)-6-hydroxy-2,2,7,8-tetramethylchroman-2-carboxylic acid (Trolox^®^); linoleic acid; chlorogenic acid hemihydrate; gallic acid monohydrate; and quercetin trihydrate were purchased from Sigma-Aldrich Inc.(Germany/USA). HPLC grade solvents, MeCN and H_3_PO_4_ were used for HPLC analyses and were obtained from Merck (Germany). For other analyses redistilled water and analytical grade chemicals and solvents (POCh S.A., Poland) were used.

Absorbance was measured using a Lambda 25 spectrophotometer (Perkin-Elmer, USA), in 10 mm quartz cuvettes. Samples were incubated in a constant temperature using a BD 23 incubator (Binder, Germany). HPLC analyses were carried out on a Waters 600E Multisolvent Delivery System (Waters Co., USA) with a PDA detector (W 996), a 20 μL sample injector (Rheodyne 7725 i), and a LC workstation equipped with Waters Empower software for data collection and acquisition. A C18 Lichrosphere 100 column (5 μm, 250 mm × 4.6 mm, i.d.) (Merck) guarded by a C18 Hypersil ODS pre-column (5 μm, 4 × 4 mm, i.d.; Agilent Technologies, USA) was used. Constant temperature of the column was maintained using a Jetstream Plus 5480 termostat (Peltier, Austria). Before injection to HPLC system, samples were filtered through a PTFE syringe filter (13 mm, 0.2 µm, Whatman, USA).

### 3.3. Preparation of plant extracts for testing antioxidant activity and phenolic profile

The samples of plant material were air-dried under normal conditions, powdered with an electric grinder, and sieved through a 0.315-mm sieve. An accurately weighed mass (2.0 g for the LA-peroxidation test, and 100–250 mg depending on the plant tissue analysed, for the other assays) was refluxed first for 30 min with 70% (v/v) aqueous MeOH (30 mL) [4], and then twice for 15 min with more of the same solvent (20 mL). The obtained extracts were combined, filtered and diluted with MeOH to 100 mL to give the test extracts (TE).

### 3.4. DPPH free radical-scavenging test

The scavenging activity was determined based on the method of Brand-Williams, Cuvelier, and Berset [26] with slight modifications. The DPPH working solution (35.5 mg/L, 90 μM) was prepared in methanol and equilibrated everyday to the absorbance of the negative control of 0.700 ± 0.030 at 517 nm. It was prepared by mixing the DPPH working solution (2 mL) with methanol (1 mL). Five different concentrations of all extracts and standards were prepared in methanol-water (70:30, v/v). An aliquot of the sample (1 mL) was added to the equilibrated DPPH working solution (2 mL) and vigorously shaken. After 60 min of incubation in screw-cap vials at room temperature in the dark, the decrease in the absorbance was measured at 517 nm. The mixtures of the sample (1 mL) and methanol (2 mL) were used as blanks. The concentration of the analyte (standard or plant material used for extract preparation) in the reaction medium (in μg/mL) was plotted against the percentage of remaining DPPH using the DPPH calibration curve, and the EC_50_ value was calculated. The standards of quercetin and trolox were used as positive controls. The activity of the plant materials was then expressed in terms of trolox (TEAA) equivalent antioxidant activity in mmol/g dw.

### 3.5. Linoleic acid (LA) peroxidation test

The ability of TE to inhibit AAPH-induced LA-peroxidation was assayed according to the method of Azuma *et al*. [27] with some modifications. An aliquot of TE (0.30 mL) was placed in a screw-cap vial and mixed with 1.3% (w/v) LA in MeOH (1.40 mL), 0.2 M phosphate buffer (pH 7.0, 1.40 mL), and water (0.70 mL). The negative control was prepared using MeOH (0.30 mL) instead of the sample. Peroxidation was initiated by the addition of 46.35 mM AAPH solution in phosphate buffer (0.20 mL). The vial was incubated at 50.0 ± 0.1 °C in the dark, sampling being carried out every hour for up to at least 5 h until the absorbance of the control reach the maximum value. The degree of oxidation was measured according to the ferric thiocyanate (FTC) method [28,29]. The reaction mixture (0.10 mL) was diluted with 75% (v/v) MeOH (9.70 mL) and mixed with 20 mM FeCl_2_ solution in 3.5% (w/w) HCl (0.10 mL) and 10% (w/w) aqueous NH_4_SCN solution (0.10 mL). After precisely 3 min the absorbance was measured at 500 nm versus 75% MeOH. Possible interference of phenolic extracts with the FTC assay was investigated by adding an extract sample to the fully oxidised negative control and no interference was observed. The inhibition ratio (*I%*) of the peroxidation process was calculated as follows: *I% = 100* *× (1−ΔA_sample_/ΔA_control_)*, where *ΔA* is the difference between the absorbance measured at the end and the start of the test.

### 3.6. Determination of total phenolic content

The total phenolic content in TE was determined according to Folin-Ciocalteu (FC) method [3]. Results were expressed as gallic acid (GAE) equivalents per dry weight of the plant material.

### 3.7. Determination of total proanthocyanidin content

The total proanthocyanidin content in TE was quantified by the modified acid/butanol assay [30]. An aliquot of TE (0.5 mL) was placed in a screw-cap vial and mixed with *n*-BuOH-35% HCl (95:5, v/v, 3 mL) and 2% (w/v) NH_4_Fe(SO_4_)_2_ × 12 H_2_O in 2 M HCl (0.1 mL). After 45 min of incubation at 95.0 ± 0.2 °C the vial was cooled to 25 °C, and the absorbance was read at 550 nm versus the unheated sample used as the blank. The results were expressed as cyanidin chloride (CyE) equivalents per dry weight of plant material.

### 3.8. Total flavonoid content

The content of flavonoids in TE was determined by RP-HPLC as the total content of flavonoid aglycones after acid hydrolysis, according to the method optimized and validated previously [31].

### 3.9. The content of chlorogenic acid isomers

The content of main caffeoylquinic acids in TE was estimated by RP-HPLC. The samples of TE were filtered through a syringe filter, and the filtrate was injected (20 µL) into the HPLC system. The elution system consisted of solvent A (0.5% water solution of ortophosphoric acid, w/v) and solvent B (MeCN) with the elution profile as follows: 0-1 min, 5% B (v/v); 1-4 min, 5-15% B; 4-10 min, 15% B; 10-11 min, 15-50% B; 11-15 min, 50% B; 15-16 min, 50-5% B; 16-22 min, 5% B (equilibration). All gradients were linear. The flow rate was 1.2 mL/min, the column was maintained at 40 °C, and the detection wavelength was set at 350 nm. Two main chlorogenic acid isomers in TE were identified by comparison with the standards of 5-*O*-caffeoylquinic acid (chlorogenic acid, ChA, commercial standard) and 3-*O*-caffeoylquinic acid (neochlorogenic acid, NChA, qualitative standard prepared by isomerisation of ChA using the method of Nagels *et al.* [32]). The contents of the two isomers were expressed per dry weight of the plant material and were calculated from the calibration curve of ChA.

### 3.10. Statistical analysis

The statistics (calculation of standard deviation, analysis of variance, partitional and cluster analysis) were performed using the software StatisticaPl for Windows (StatSoft Inc., Poland).

## 4. Conclusions

The present study demonstrated that the studied plant materials possess significant and dose-dependent *in vitro* radical-scavenging activity towards the DPPH radical and a strong ability to inhibit the AAPH-induced oxidation of linoleic acid, both of which correlate with their polyphenolic composition. Given the remarkably high phenolic content found in some tested materials (samples grouped in the cluster CL1), this study supports the idea that *Sorbus* products may be good sources of powerful natural antioxidants for use in food, medicine, cosmetics and other fields that require antioxidants. However, further research is needed to investigate the structure and antioxidant activity of the individual phenolic compounds constituting the analysed extracts. Further studies would also be desirable to clarify the possible toxicity and other biological properties of the species presented here; moreover, the effects of the use of these natural antioxidants on food, cosmetic or drug sensory properties (such as odour and taste) should be addressed in future research.
